# Clinical Images: Sarcoidosis revealed by recurrent dactylitis

**DOI:** 10.1002/art.43355

**Published:** 2025-11-30

**Authors:** Thomas Subervie, Thibault Willaume, Amin Maazouzi, Jacques‐Eric Gottenberg, Jean Sibilia, Noëlle Weingertner, Eden Sebbag, Marc Scherlinger

**Affiliations:** ^1^ Strasbourg University Hospital Strasbourg France; ^2^ National Center for Rare Autoimmune Systemic Diseases Est Sud‐Ouest, Strasbourg University Hospital Strasbourg France; ^3^ National Center for Rare Autoimmune Systemic Diseases Est Sud‐Ouest, Strasbourg University Hospital and UMR_S INSERM 1109, Immunorhumatologie Moléculaire Strasbourg France

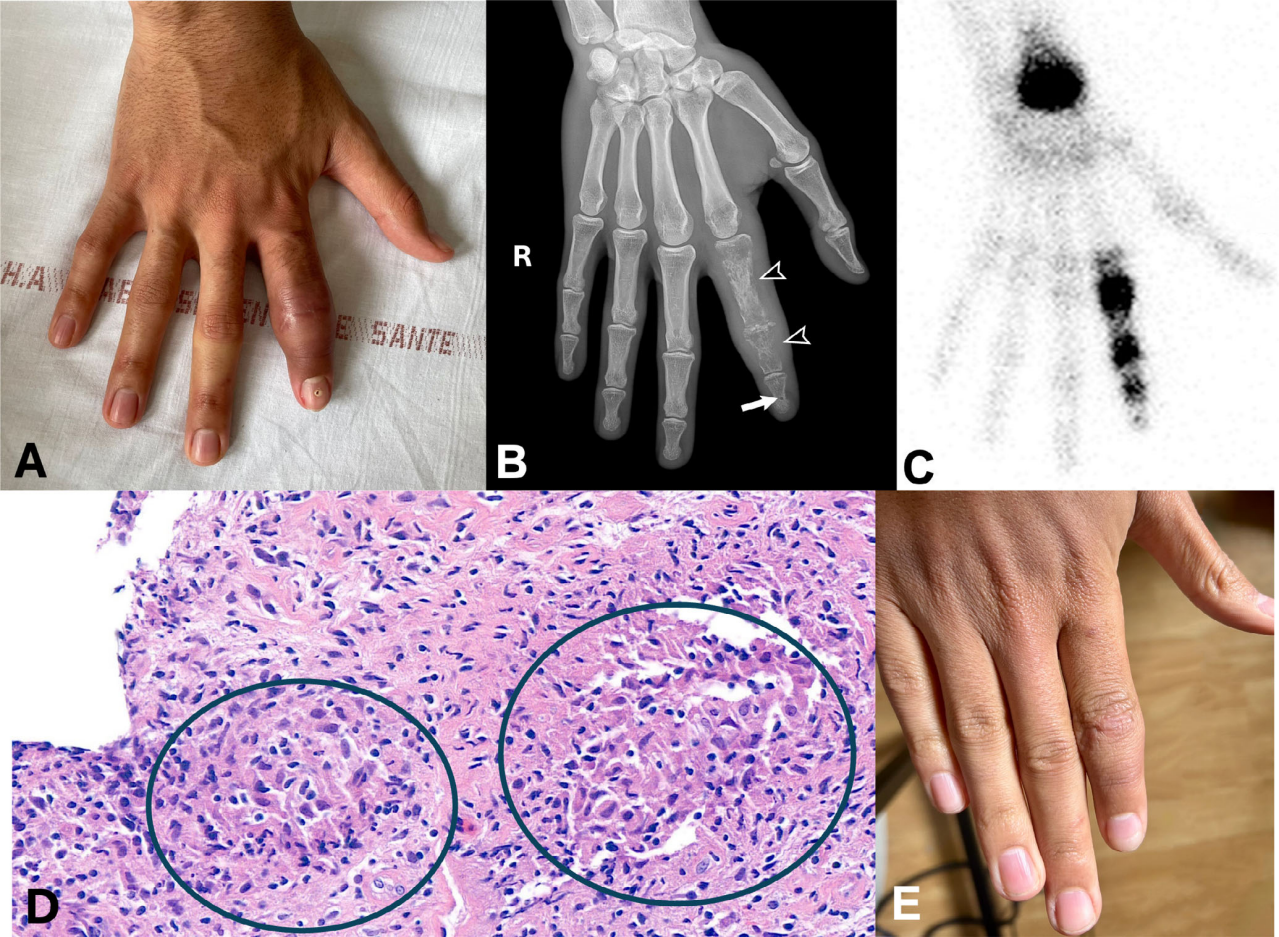

(A) A 21‐year‐old man presented with chronic and recurrent dactylitis of the index finger. (B) The conventional radiography showed osteolytic changes in the three phalanges of the right index finger, with a characteristic honeycomb (or lattice) appearance in P1 and P2 (black arrowheads) and a cystoid lesion in P3 (white arrow) associated with thickening of the surrounding soft tissues. Of note, there is no joint space narrowing in the metacarpophalangeal and interphalangeal joints. (C) Technetium‐99 scintigraphy identified multiple areas of increased uptake. (D) Histopathological analysis of biopsies obtained by a hand surgeon showed chronic aseptic osteitis and noncaseating granulomas (green circles) within the synovium, consistent with a diagnosis of sarcoidosis. Interferon‐γ release assay for *Mycobacterium tuberculosis* was negative, as was culture for mycobacteria and *M tuberculosis* polymerase chain reaction. Additional findings supporting the diagnosis of systemic sarcoidosis included hypermetabolic mediastinal and hilar lymphadenopathies on positron emission tomography–computed tomography with pulmonary micronodules, elevated angiotensin‐converting enzyme, hypergammaglobulinemia, and lymphopenia. Bone involvement may be present in 1% to 15% of patients with sarcoidosis and is often asymptomatic.^1^ The spine is the most commonly affected site, followed by the pelvis.^2^, ^3^ The classic lesions in the small bones of the hands and feet like in this case are known as Perthes and Jüngling disease.^2^ (E) The patient was treated with a tapering course of glucocorticoids and methotrexate, and there was improvement in the dactylitis.

## Supporting information


Disclosure Form
